# Modern and historical uses of plant grafting to engineer development, stress tolerance, chimeras, and hybrids

**DOI:** 10.1111/tpj.70057

**Published:** 2025-02-21

**Authors:** Frauke Augstein, Charles W. Melnyk

**Affiliations:** ^1^ Department of Plant Biology Swedish University of Agricultural Sciences Uppsala Sweden

**Keywords:** plant grafting, mobile RNAs, dwarfing, development, stress tolerance, graft hybrids, graft chimeras

## Abstract

For millennia, people have grafted plants to propagate them and to improve their traits. By cutting and joining different species or cultivars together, the best properties of shoot and roots are combined in one plant to increase yields, improve disease resistance, modify plant growth or enhance abiotic stress tolerance. Today, grafting has evolved from what originated as an early form of trait engineering. The fundamental technique remains the same, but new species are being grafted, new techniques have developed and new applications for modifying development and stress tolerance are appearing. In addition, engineering possibilities such as graft chimeras, graft hybrids and the use of mobile RNAs are emerging. Here, we summarize advances in plant grafting with a focus on engineering novel traits. We discuss traditional uses of grafting to engineer traits but also focus on recent developments, challenges and opportunities for plant improvement through grafting.

## Significance Statement

Plant grafting has been practiced for millennia and recent developments have resulted in new species, new techniques and new applications appearing. Here, we review these advances focusing on developmental changes, stress tolerance, graft chimeras, graft hybrids, and mobile RNAs systems that use graft engineering to modify and improve the phenotypes of plants.

## INTRODUCTION

The cutting and joining of different plants is a common horticultural practice that has evolved over millennia involving a shoot (scion) grafted to a root or stem (rootstock). It remains unknown when people first started grafting but they were likely inspired by nature where stems and roots of many woody species commonly fuse within and between plants. Such a process is surprisingly common and at least 200 tree species graft their roots to one another, and in some forests, up to 75% of trees are naturally grafted to one another via their roots (Külla & Lõhmus, 1999; Lev‐Yadun & Sprugel, 2011). Early grafters may have tied stems together to allow branches to fuse, a process known as inosculation, or cut and joined stems together to heal as one. Grafting of woody plants allowed desirable shoots to be asexually propagated, providing an early means for people to domesticate fruit trees (Mudge et al., 2009). Ancient texts also describe grafting of species that naturally root well (Mudge et al., 2009) suggesting that some unknown benefit may have been obtained from grafting rather than taking cuttings. By the 15th century, desirable scions were grafted to dwarf apple rootstocks (Mudge et al., 2009). These plants may have been some of the first whose traits were engineered or modified through grafting. Modifying plants through grafting presented several important advantages. Firstly, there was no longer a need for long breeding cycles to introduce new traits, instead, benefits such as dwarfing could be rapidly induced through grafting. A second advantage was the ability to combine traits from species that could not be crossed together, or traits for which the genetics are complex and not based on a single gene. Finally, grafting separated properties of scion and rootstock, allowing each to be bred separately for desirable traits. Thus, a technology was developed that continuously evolves. Today, plants are still grafted for asexual propagation, particularly in forestry and horticulture, but far more plants are being grafted to engineer their properties (Garner & Bradley, 2013; Lee et al., 2010). Here, we discuss how grafting modifies a plant's properties, how humans have used this to engineer plants, and present challenges and opportunities associated with engineering plants through grafting (Boxes 1 and 2).

Box 1 **Main points**

1. Grafting improves tolerance against both biotic and abiotic factors by grafting stress‐resistant rootstocks to high yielding or enhanced fruit quality scions.
2. Grafting causes major change in plant development including increasing or decreasing plant size, accelerating flowering time or changing the perceived age of grafted tissues.
3. Graft chimeras, a mixture of cells from two species, can arise from the graft junction to form chimeric plants with the potential to improve disease resistance, fruit quality, and plant growth.
4. Graft hybrids form at the graft junction from the exchange of DNA between cells, and upon the isolation and regeneration of these cells, allows asexual hybridization between graft compatible plants.
5. RNA mobility occurs between scion and rootstock, and when mobile CRISPR‐Cas9 or RNA silencing transgenes are introduced to rootstocks, they can induce heritable gene editing in the scion or confer systemic resistance to pathogens, respectively.

Box 2 **Open questions in graft engineering**

1. Can we deploy grafting in forestry and more widely in horticulture by enhancing graft automation, improving rootstock breeding programs, and lowering the costs of grafted plants?
2. What are the genes and loci causing developmental and stress‐related grafting phenotypes, and can these loci be incorporated into rootstock breeding programs?
3. Can we identify loci responsible for graft failure or graft success, and modify their expression to greatly expand the range of grafted plants?
4. Can we develop robust techniques for graft chimera formation and use these chimeras more widely in scientific research and horticulture?
5. How does DNA transfer at the graft junction and can we use non‐transgenic means to improve hybridization efficiency and hybrid recovery?
6. How can we improve the efficiency of systemic CRISPR‐Cas9 mediate genome editing and does this technology apply to species beyond the Brassica family?

## TECHNOLOGICAL ADVANCES IN GRAFTING

The creativity of the grafter, technological innovations, and breeding efforts have driven modern grafting, and here, we discuss these three aspects. Ancient texts described extensive graft combinations, many of which we know today are unsuccessful, suggesting early grafters were highly creative making combinations for curiosity, atheistic interest or horticultural advantage. Several innovations are particularly noteworthy. Firstly, the use of dwarfing rootstocks in fruit production increased yields and facilitated harvesting due to shorter and more densely planted trees (Garner & Bradley, 2013; Lordan et al., 2019). This may have been practiced in ancient China when kumquat or mandarin scions were grafted to trifoliate orange rootstocks, a dwarfing combination, but the precise date and whether this combination was done for dwarfing is unknown (Mudge et al., 2009). In the 1400s, grafting was done with a weak growing apple tree as a rootstock, the Paradise apple, that caused the scion to grow much smaller than normal (Mudge et al., 2009). This began a practice of dwarfing that quickly gained popularity. The phylloxera insect's arrival from North America precipitated another round of creativity when European grapes used for wine production had no resistance and were dying from insect attack. Initial efforts centered on pest control or hybridization, but the most suitable and durable solution emerged from grafting American grape rootstocks to European grape scions. This conferred resistance to soil infections while maintaining European grape berries thus rescuing the wine industry (Mudge et al., 2009). A third creative innovation was the introduction of vegetable grafting. Early records from China indicated that bottle gourd grafting was practiced in the 6th century, and much later, in the 1920s, Japanese researchers grafted squash rootstocks to watermelon to reduce fungal wilt. However, vegetable grafting became more commonly practiced in the 1950s when greenhouse agriculture became more prevalent and the density of pests and reluctance to use pesticides strengthened (Lee et al., 2010). Today, many greenhouse‐grown tomatoes, cucumbers, melons, eggplants, and gourds are grafted to disease resistance rootstocks. By some estimates, over 1 billion vegetables are grafted yearly (Lee et al., 2010). Future creative efforts in grafting will likely also play important roles (Table 1). For instance, forestry has largely avoided grafting with exceptions for breeding and seed production (Jayawickrama et al., 1991), yet with improved rootstocks and an automated grafting process, grafting could provide major benefits to the forestry industry (Box 2).

**Table 1:** Developments in graft innovation

Advance	Time	Innovation
Propagation	Antiquity	Grafting is used for the asexual propagation of desirable fruit trees (Mudge et al., 2009)
Dwarfing	1472	The Paradise dwarfing apple is first mentioned in grafting literature, likely as a rootstock for dwarfing (Mudge et al., 2009)
Graft chimeras	1674	The ‘Bizzaria’ graft chimera is mentioned. In 1825, the + *Laburnocytisus* ‘*Adamii*’ chimera appears. In 1907, the first purposely made chimeras appear (Frank & Chitwood, 2016; Mudge et al., 2009)
Phylloxera resistance	1869	Grafting is proposed as a solution for grapevines to resist phylloxera (Mudge et al., 2009)
Interstock grafting	1900s	Interstock grafting was likely practiced before the 1900's, but became more common with pear in the 1930s as a method for dwarfing with quince rootstocks (Grubb, 1939; Hatton, 1939)
Vegetable grafting	1920s	Research began in the 1920s, and by the 1930s grafted vegetables were used in agriculture (Sakata et al., 2007)
Micrografting	1950s	Developed in the 1950s for ivy and chrysanthemum, later applied in the 1970s to generate virus‐free citrus (Jonard, 1986)
Robot automation	1987	The first semi‐automated grafting robots were developed (Lee et al., 2010)
Graft hybrids	2009	Using transgenic Nicotiana, hybrid cells are recovered and grown to mature plants. In 2014, novel hybrid species are formed (Fuentes et al., 2014; Stegemann & Bock, 2009)
Monocot grafting	2022	Originally proposed by Obolonsky in 1960, embryo transplantation methods are developed for efficient monocot grafting (Obolensky, 1960; Reeves et al., 2022)

Importantly, creative efforts have worked together with technological and breeding efforts. Technology and techniques have allowed new ways to graft plants. Experienced grafters are aware of the importance of the grafting method and timing. Over millennia, techniques such as bud grafting, veneer grafting, splice grafting, side grafting, tongue‐and‐whip grafting, approach grafting and cleft grafting were developed to graft different tissues and species (Garner & Bradley, 2013). Time of the year is also important for grafting. For instance, grape vines, cherries and apples are often grafted in late winter, whereas other species such maples, mulberries, and walnuts graft well in the autumn (Garner & Bradley, 2013). Widespread grafting of new species such as vegetables promoted the development of grafting methods such as hole insertion grafting, pin grafting and tongue approach grafting (Lee et al., 2010). In forestry, top grafting of juvenile branches to mature branches began in the 1970s to accelerate the flowering time of conifer scions and to speed up conifer breeding cycles, practices still used today with conifers and more recently with eucalyptus (de Oliveira Castro et al., 2021; Heuchel et al., 2024). Micrografting, the process of grafting very small or even microscopic tissues, was developed in the 1950s for ivy and chrysanthemum and was later applied in the 1970s to eliminate viruses from citrus trees (Jonard, 1986; Murashige et al., 1972). Today, micrografting is more commonly used in research to graft small plants such as Arabidopsis or to graft seedling material (Bartusch & Melnyk, 2020; Feng et al., 2024; Reeves et al., 2022). Micrografting with conifer trees allowed the successful formation of pine‐spruce grafts, something not previously possible with traditional grafting techniques (Feng et al., 2024). An important milestone was the development of embryonic grafting that allowed the successful grafting of monocots, previously considered ungraftable (Reeves et al., 2022). Embryonic grafting allowed success within a species but also between different monocot species and genera (Reeves et al., 2022). The automation of grafting with semi‐automatic robots is another notable technology advance yet grafting rates are only double what can be done manually (Xie et al., 2020). The higher costs of robots means they are not being widely deployed and manual grafting remains popular (Lee et al., 2010; Xie et al., 2020). Other innovations, such as the hot callus method of applying heat locally to the graft junction have dramatically improved graft formation rates in some recalcitrant species such as walnut (Avanzato & Tamponi, 1988). Applying enzymes and hormones to the graft junction can also improve graft formation rates (Kawakatsu et al., 2020; Köse & Güleryüz, 2006).

**Figure 1:**
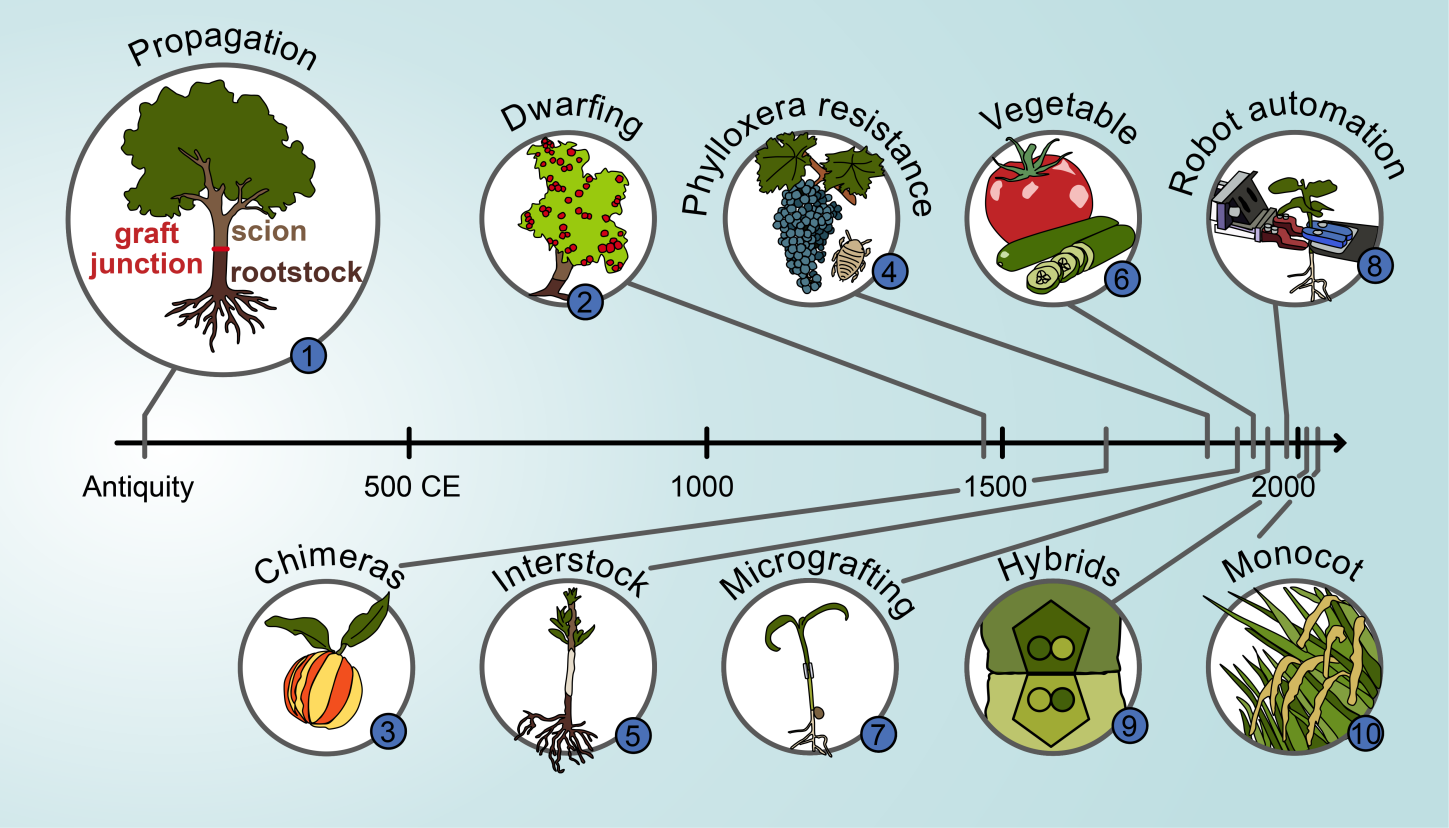
Developments in graft innovation. From the antiquity to today, several innovations have expanded our use of grafting to combine desirable traits of two plants. The ten events presented here are further described in Table 1. The axis represents time in the Common Era (CE).

Breeding efforts of rootstocks have also played a critical role in the development and widespread use of grafting (Warschefsky et al., 2016). Dwarfing rootstocks for apples emerged in the 1400s, and several hundred years later, many different rootstocks are available with different dwarfing strengths as a result of breeding efforts (Garner & Bradley, 2013; Mudge et al., 2009). Thus, a farmer can tailor their orchard height by selecting the correct rootstock. With grapevine rootstocks, breeding efforts have gone into crossing together different American grape varieties together to obtain plants that are both disease resistant but also suitable for different soil types (Rahemi et al., 2022). Although common European grapes varieties are largely unchanged for hundreds of years, many popular rootstocks were developed in the late 19th or early 20th century, and breeding is ongoing today (Ollat et al., 2016). In addition to apples and grapevines, breeding is done on a variety of both vegetable and woody rootstocks to improve yields, abiotic stress tolerance and disease resistance (Warschefsky et al., 2016). Through a combination of creativity, technology, and breeding, there is a strong potential to lower the cost of grafting, enhance the benefits of grafted plants and to widen the range of species grafted (Figure 1; Table 1).

## HOW GRAFTING AFFECTS PHENOTYPES

**Figure 2:**
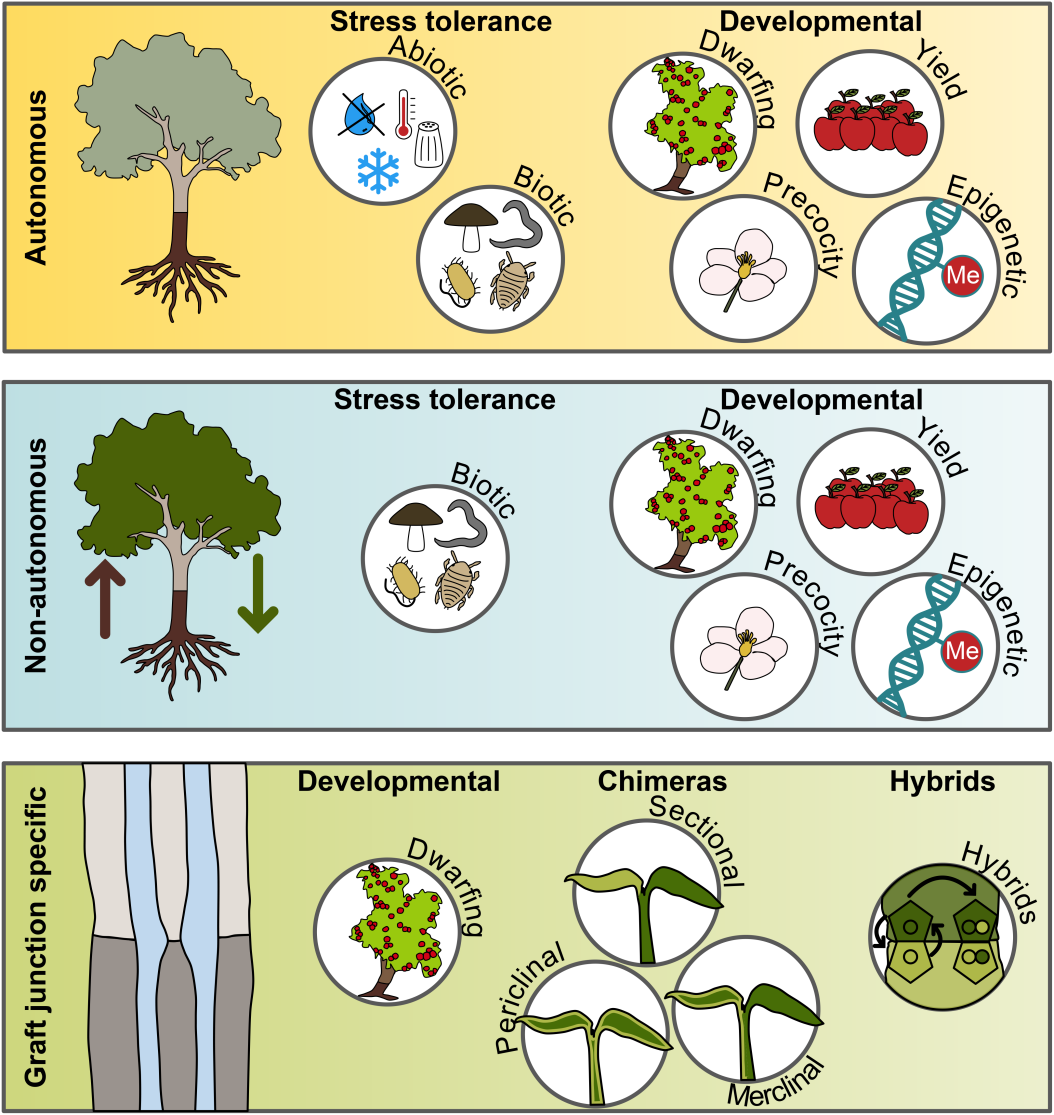
Grafting's effect on plant phenotypes and their impact on engineering. Grafting can affect the plant's phenotype autonomously, non‐autonomously through transfer of components between scion and rootstock, or due to effects at the graft junction. Desired phenotypes are often related to stress tolerance, development or the formation of chimeras or hybrids.

Grafting can modify plant phenotypes in three general ways (Figure 2). There can be autonomous changes that are inherent to the scion or rootstock, such as a rootstock that is resistant to a soil‐borne disease thus providing whole plant resistance. Secondly, the graft junction itself can modify phenotypes. Graft junctions often have disorganized vascular morphology and some dwarfing rootstocks affect xylem formation and vascular connectivity (Olmstead et al., 2006; Soumelidou et al., 1994), potentially affecting vascular transport. Graft junctions can also be enriched in non‐differentiated cells, contributing to weakening of the stem (Errea et al., 1994). Hence, some rootstocks are probably only partially compatible and their failure to fully reconnect the stem could contribute to changing scion growth phenotypes. Finally, and probably the best studied, graft‐induced phenotypes can be non‐autonomous and caused by an interaction between scion and rootstock, often related to mobile substances.

In principle, hundreds, thousands, or even millions of molecules are exchanged between shoot and root. When different genotypes are grafted together, this flow of molecular information between scion and rootstock is modified. In the recipient tissue, novel molecules can appear or molecules already present in the tissue can increase or decrease in levels. Plants have extensive vascular networks that communicate and exchange RNAs, proteins, metabolites, hormones, and sugars within organs and between shoot and root (Lucas et al., 2013). Successful grafting requires the reconnection of the sugar‐transporting tissues, the phloem, and the water‐conducting tissues, the xylem (Melnyk et al., 2015). Using grafting in research has uncovered that thousands of small RNAs including siRNA and miRNAs are mobile from shoots to roots in a variety of species including Arabidopsis, grapevines, legumes, potatoes and Nicotiana (Bhogale et al., 2013; Li, Wang, et al., 2021; Pant et al., 2008; Rubio et al., 2022; Zhang et al., 2021). Notably, small RNA movement follows a source to sink route consistent with transport through the phloem. Multiple papers have also reported that hundreds or thousands of mRNA transcripts move between shoots and roots in Arabidopsis, legumes, potato, datura and apple (Li et al., 2024; Li, Wang, et al., 2021; Notaguchi et al., 2014; Thieme et al., 2015; Zhang et al., 2022). Mobility appears related to transcript abundance in the phloem (Calderwood et al., 2016), RNA modifications (Yang et al., 2019) or the presence of certain tRNA motifs (Zhang et al., 2016). However, identifying mobile RNAs relies on polymorphisms between scion and rootstock genomes to understand where the transcript originated from. A recent study found that many annotated mobile mRNA transcripts lack support from reanalyzed RNAseq data due to biological variation, technological noise, and incomplete genome assemblies (Paajanen et al., 2024). Thus, the issue of mRNA mobility warrants further investigation and more thorough use of long read sequencing and accurate polymorphism annotation. Hormones, including the jasmonic acid precursor OPDA, the gibberellic acid precursor GA12, and cytokinins also move across the graft junction, as do metabolites including glucosinolates (Andersen et al., 2013; Dong et al., 2022; Matsumoto‐Kitano et al., 2008; Regnault et al., 2015; Schulze et al., 2019). Likely hundreds of proteins are mobile between scion and rootstock, though efforts to identify these on a genome‐wide level are more limited due to the sensitivity of mass spec and a reliable way to identify whether the protein came from the scion or rootstock. Grafting studies helped establish that the flowering time regulator FT protein moves from the leaves to the apical meristem (Corbesier et al., 2007). Further work is needed to uncover the long distance mobile proteome in plants.

Importantly, these studies discovered that grafted plants exchange numerous molecules that can affect traits. Such exchanges are likely the basis for many of the observed grafting‐induced phenotypes and the grafting‐induced expression changes in hundreds or thousands of genes in grafted grapevine, watermelon, bottle gourd, tomato, potato, or datura (Cookson & Ollat, 2013; Liu et al., 2016; Wang et al., 2019; Zhang et al., 2022). European grape vines are commonly grafted to American grape rootstocks which affects volatiles, phenolics, and esters in the wine (Chen et al., 2024; Ollat et al., 2003). Some changes in gene expression may be caused by the grafting process itself in Arabidopsis (Kumari et al., 2015), consistent with the notation that the junction plays some role in graft‐induced phenotypes. Altogether, thousands or millions of molecules are moving within a plant, and by grafting different plants together, we can change the abundance of molecules and also their composition, thus providing a novel source of phenotypic change.

## STRESS TOLERANCE

Grafting is widely used to improve biotic and abiotic stress tolerance (Figure 2). One of the first uses was conferring resistance in grapevine against the insect phylloxera that attacks roots. Today, the majority of grafted plants are vegetables and there, grafting is primarily done in tomato, eggplant, pepper, watermelon, melon, and cucumber to control soil‐related pests such as nematodes, bacteria, fungi, or insects. Commercial vegetable grafting began in Asia the 1930s in response to diseases such as fusarium wilt resulting from the continuous cropping of plants (Sakata et al., 2007). More recently, the fungicide methyl bromide was banned in 2005, decreasing the tools available for fungal control and increasing the use of grafted vegetables in Europe and North America (Cohen et al., 2007). New strains of fusarium have also appeared that overcome host plant resistance necessitating grafting until new cultivars become available (King et al., 2008). Altogether grafting for disease resistance is growing in popularity as diseases emerge, pesticide options reduce and cropping systems become more intensive.

In watermelon and melons, grafting primarily provides resistance to fusarium wilt but can also help with resistance to nematodes and verticillium fungus (King et al., 2010). Grafting in cucumbers provides resistance to fusarium wilt (King et al., 2010), while grafting late blight resistance potato rootstocks increases resistance in susceptible potato scions (Li & Zhao, 2021). Tomatoes are commonly grafted in open field production and non‐heated greenhouses for improved disease resistance (King et al., 2010). Most disease‐resistant tomato rootstocks are hybrids between tomato (*Solanum lycopersicum*) and *Solanum habrochaites* (King et al., 2010). Two common tomato rootstocks, ‘Beaufort’ and ‘Maxifort’, are resistant to pathogens such as Tomato Mosaic Virus, fusarium root rot and fusarium crown rot, corky root, verticillium, and nematodes (King et al., 2010). Grafted tomato rootstocks can provide good resistance to bacterial wilt (ralstonia) with no losses compared to non‐grafted controls that suffer between 30 and 80% disease incidence (Suchoff et al., 2019). The basis for such fungal, bacterial, nematode, and insect resistance appears largely autonomous and restricted to the root though there are several examples showing that resistance might be more systemic, perhaps due to mobile substances or a general strengthening the vigor of the scion allowing it to better resist infection (Lee, 1994). In apples, fireblight (Erwinia) bacterial resistance in the scion can be modified depending on the rootstock genotype but this might be related to dwarfing (Korba et al., 2002; Singh et al., 2019). With plant viruses, many move systemically and here, the rootstock can increase resistance in the scion due to small RNAs moving from rootstock to scion and promoting RNA silencing from systemic mobile RNAs (Spanò et al., 2015, 2020). Engineering rootstocks to produce transgenic small RNAs can confer resistance to the scion such as towards potato spindle tuber viroid in Nicotiana or towards Prunus necrotic ringspot virus in cherry trees (Kasai et al., 2013; Zhao & Song, 2014).

Grafting for abiotic stress tolerance is also common. In vegetables, cold tolerance is one of the major reasons for grafting cucumber scions to gourd rootstocks. Gourd rootstocks are much more tolerant of cold soil temperatures when grafted to cucumber scions, improve their cold tolerance (King et al., 2010). Tomato scions grafted to *Solanum peruvianum* rootstocks showed improved heat stress tolerance (Lee et al., 2023), while tomato scions grafted to *Solanum habrochaites* rootstocks showed improved cold tolerance (Ntatsi et al., 2017). Grafting tomato scions to salt or drought‐tolerant rootstocks is also a promising method for increase tolerance to these stresses (Singh et al., 2020; Zhang et al., 2019). In citrus, resistant rootstocks can improve heat, drought or cold tolerance of sensitive scions (Balfagón et al., 2022; Hmmam et al., 2023). Altogether, the use of stress tolerant rootstocks has great potential but for many traits, the mechanistic basis for tolerance is not well known and may rely on improved properties of the rootstock tolerating root‐specific conditions, or overall improvements to growth and vigor that helps tolerate the stress.

## DEVELOPMENTAL CHANGES

Grafting changes the way plants grow and develop (Figure 2). This is particularly evident from the widespread use of dwarfing rootstocks, first identified in the 1400s, that now are commonly used with apples, cherries, pears, plums, peaches, and many other soft fruits (Garner & Bradley, 2013). The rationale is that by using rootstocks that restrict tree growth, trees can be planted more densely, increasing yields per unit area and facilitating harvesting (Garner & Bradley, 2013; Lordan et al., 2019). Interestingly, dwarfing rootstocks can also cause trees to produce fruits at an earlier age and also increase yields compared to a non‐dwarfing tree of similar size (Seleznyova et al., 2008). Thus, there is considerable interest in identifying the molecular basis for dwarfing. Quantitative trait loci (QTL) studies identified three loci in apples responsible for dwarfing: *Dw1* on chromosome 5, *Dw2* on chromosome 11 and *Dw3* on chromosome 13 (Foster et al., 2015; Harrison et al., 2016). *Dw1* has the strongest effect while *Dw2* alone does not induce dwarfing (Foster et al., 2015). A candidate gene approach tested WRKY expression in dwarfing rootstocks and found *WRKY9* expression was elevated, and by overexpressing this gene, dwarfing could be induced (Zheng et al., 2018). However, this study did not correlate *WRKY9* with *Dw1*, *Dw2*, or *Dw3*. A QTL approach focused on *Dw1* in apple and found a transposable element inserted upstream of *AUXIN RESPONSE FACTOR3* (*ARF3*) located in the region of *Dw1* (Li et al., 2024). Expression of *ARF3* was reduced in dwarfing rootstocks, yet key experiments knocking out this gene in apples to confer dwarfing were missing, leaving uncertainty whether this transposable element in the *ARF3* promoter is the causative factor of *Dw1* (Li et al., 2024). Carbohydrate depletion and a reduction in polar auxin transport have also been suggested as possible reasons for dwarfing (Foster et al., 2017). In addition, dwarfing rootstocks have lower hydraulic conductivity and less potential to transport water, another possible contributing factor (Atkinson et al., 2003). Thus, further work is needed to uncover or validate the identity of *Dw1*, *Dw2*, and *Dw3* and to understand the mechanism for dwarfing. Interestingly, in pear trees, the dwarfing locus *Dw1* appeared to be in a similar chromosome location as *Dw1* in apple, suggesting a similar locus or gene might be responsible for dwarfing in both species (Knäbel et al., 2015).

In contrast to trees, grafting is used in some vegetable crops to increase vigor and enhance yields per individual plant. Grafted greenhouse tomatoes can be more vigorous, halving the number of plants grown and reducing seed costs (King et al., 2010). Grafting with certain rootstocks can increase vigor and yields in eggplant and double yields in tomato (Grieneisen et al., 2018; Musa et al., 2020). Grafting a cassava relative, *Manihot glaziovii*, as a scion onto *Manihot esculenta* (cassava) roots can double yields compared to ungrafted cassava (Bruijn & Dharmaputra, 1974). However, too much vigor in tomato can come at the expense of fruit yields (King et al., 2010) and can also deteriorate fruit quality in watermelons grafted to bottle gourd (Garcia‐Lozano et al., 2020). The molecular basis for such changes in vigor are not well known, but are likely related water and nutrient update, mobile substances from the rootstock, or changes in gene expression in the scion (Kyriacou et al., 2017). Rootstocks with increased xylem cell widths were associated with higher yields in grafted eggplants (Kappel et al., 2024). Decreases in DNA methylation were also observed in grafted eggplants, correlating with changes in transposable element expression, suggesting that epigenetic factors might also play a role in changes in vigor (Cerruti et al., 2021). Such changes in the epigenome raise the intriguing possibility that DNA methylation changes could be inherited to the progeny. Progeny from tomato, eggplants, and pepper grafts had changes in DNA methylation compared to their parents (Wu et al., 2013). Tomato scions grafted to mutant *msh1* rootstocks showed enhanced vigor, and these improvements in vigor were heritable for up to five generations (Kundariya et al., 2020). Similarly, grafting different cultivars of pepper, *Capsicum annuum*, together caused changes in fruit size in the progeny that were heritable for several generations (Ohta & Van Chuong, 1975; Taller et al., 1998; Tsaballa et al., 2013).

Grafting can also induce changes in flowering and tissue maturity. Seminal experiments done with Arabidopsis grafting demonstrated that the FT protein, important for flowering induction, was mobile from leaves to apical meristems (Corbesier et al., 2007). Modifying FT levels in the rootstock through vernalization or using late flowering cultivars could accelerate or delay, respectively, flowering time in grafted *Brassica rapa* scions (Zheng et al., 2019). Overexpressing blueberry FT homologs in the rootstock could accelerate blueberry flowering in the scions (Song et al., 2019), while in cassava, grafting a high flowering rootstock could enhanced flowering in a scion with low flowering rates (Silva Souza et al., 2018). In several woody species, grafting a juvenile branch onto the end of a mature plant, a process known as top grafting, can accelerate flowering and help shorten breeding cycles. Eucalyptus, pine, and kiwifruit juvenile branches all flower several years earlier when grafted than would be normally expected (Almqvist & Ekberg, 2001; de Oliveira Castro et al., 2021; Liang et al., 2011). Interestingly, top grafting mature tissues repeatedly to juvenile rootstocks resulted in an opposite effect in *Sequoia sempervirens*: the restoration of juvenile traits in the scion (Huang et al., 1992). These effects of top grafting are likely promoted by mobile substances such as mRNAs and miRNAs from leaves or needles in the stock that affect scion age (Ahsan et al., 2019), or alternatively might be influenced by the vigor and carbon reserves of a mature stock that can accelerate growth and tissue maturation of the scion.

## GRAFT CHIMERAS

Grafting causes cells from both graft partners to grow together and mix. Occasionally, when tissues or organs grow from the graft junction itself, they can contain a mixture of cells from the two different species. These cells remain genetically distinct but grow as intermixed sectors or layers, known as a graft chimera. One notable example is the ‘Bizzaria’ orange discovered in 1674 from a graft of *Citrus medica* to *Citrus aurantium* (Frank & Chitwood, 2016). In 1868, Charles Darwin proposed his theory of graft hybridization whereby different parts of the grafted plant come together to create new individuals. In his book, *The Variation of Animals and Plants under Domestication*, Darwin present Adam's laburnum as an example of hybridization (Darwin, 1868; Liu, 2018), which we know today is not a true hybrid but instead a chimera of cells between *Laburnum anagyroides* and *Chamaecytisus purpureus* (Frank & Chitwood, 2016). Adam's laburnum and the ‘Bizzaria’ orange are both stable chimeras that are vegetatively propagated and still in use in the ornamental trade. In research, producing graft chimeras began in 1907 when Winkler grafted black nightshade (*Solanum nigrum*) to tomato (*S. lycopersicum*) and made a cut through the healed junction to induce adventitious shoots, some of which were graft chimeras (Winkler, 1907). Chimeras can be sectorial, which have a region of tissue of a different genotype, mericlinal, which have part of a cell layer of a different genotype or periclinal, which have an entire layer of cells in the meristem with a different genotype (Figure 2) (Jørgensen & Crane, 1927). In *Solanum* species, most chimeras that emerge from the graft junction are initially mericlinal but these are often not stable and develop into periclinal chimeras (Jørgensen & Crane, 1927).

More recently, graft chimeras have been used to understand cell layer‐specific expression patterns. Grafts between *Solanum pennellii* and tomato (*S. lycopersicum*) formed chimeras that had 382 genes mainly expressed in the L1 layer, many of these related to cutin and wax, whereas 1159 genes were expressed mainly in the L2/L3 layers, many of these related to the chloroplast (Filippis et al., 2013). Graft chimeras formed between *Citrus sinensis* and *Citrus natsudaidai* revealed the relative contribution of different cell layers to fruit and leaf characteristics (Zhou et al., 2002). The L1 layer contributed to fruit juice sacs, while the L2 layer produced seeds and the L3 layer produced vascular bundles (Zhou et al., 2002). Leaves often had intermediate characteristics, reflecting the importance of one cell layer influencing the phenotype of other layers (Zhou et al., 2002). Chimeras between *Brassica juncea* and *Brassica oleracea* also had intermediate phenotypes (Chen et al., 2006). Progeny of these plants, although non‐chimeric, showed evidence of DNA methylation and sRNAs from the other chimeric partner, suggesting cell layer exchanges of information led to heritable DNA modifications (Li et al., 2013; Yu et al., 2018). Chimeras have strong horticultural potential: by combining the best properties of different cell layers, there is an opportunity to enhance yields, fruit characteristics or disease resistance. Chimeras between *B. juncea* and *B. oleracea* increased resistance to whitefly due to cell layers from the resistant *B. oleracea* (Li et al., 2017). Graft chimeras of tomato (*S. lycopericum*) show an increased resistance to aphids when an L1 layer from *S. pennellii* was included (Goffreda et al., 1990), while graft chimeras of cassava, *M. esculenta*, with the wild species, *Manihot fortalezensis*, increased yields and improved drought tolerance (Nassar & Bomfim, 2013). Although there is strong potential to more widely study and deploy chimeras in agriculture, challenges remain with forming adventitious shoots from the graft junction and identifying chimeras. Many species are recalcitrant to shoot formation from the graft junction (Burge et al., 2002) but techniques like *in vitro* graft cultures and protoplast grafting could be alternatives to improve regeneration efficiencies (Binding et al., 1987; Noguchi et al., 1992). The recent development of callus grafting involving the co‐culturing of different calli together (Hasbioğlu et al., 2023) might also allow more efficient chimeras formation and regeneration.

## GRAFT HYBRIDIZATION

**Figure 3:**
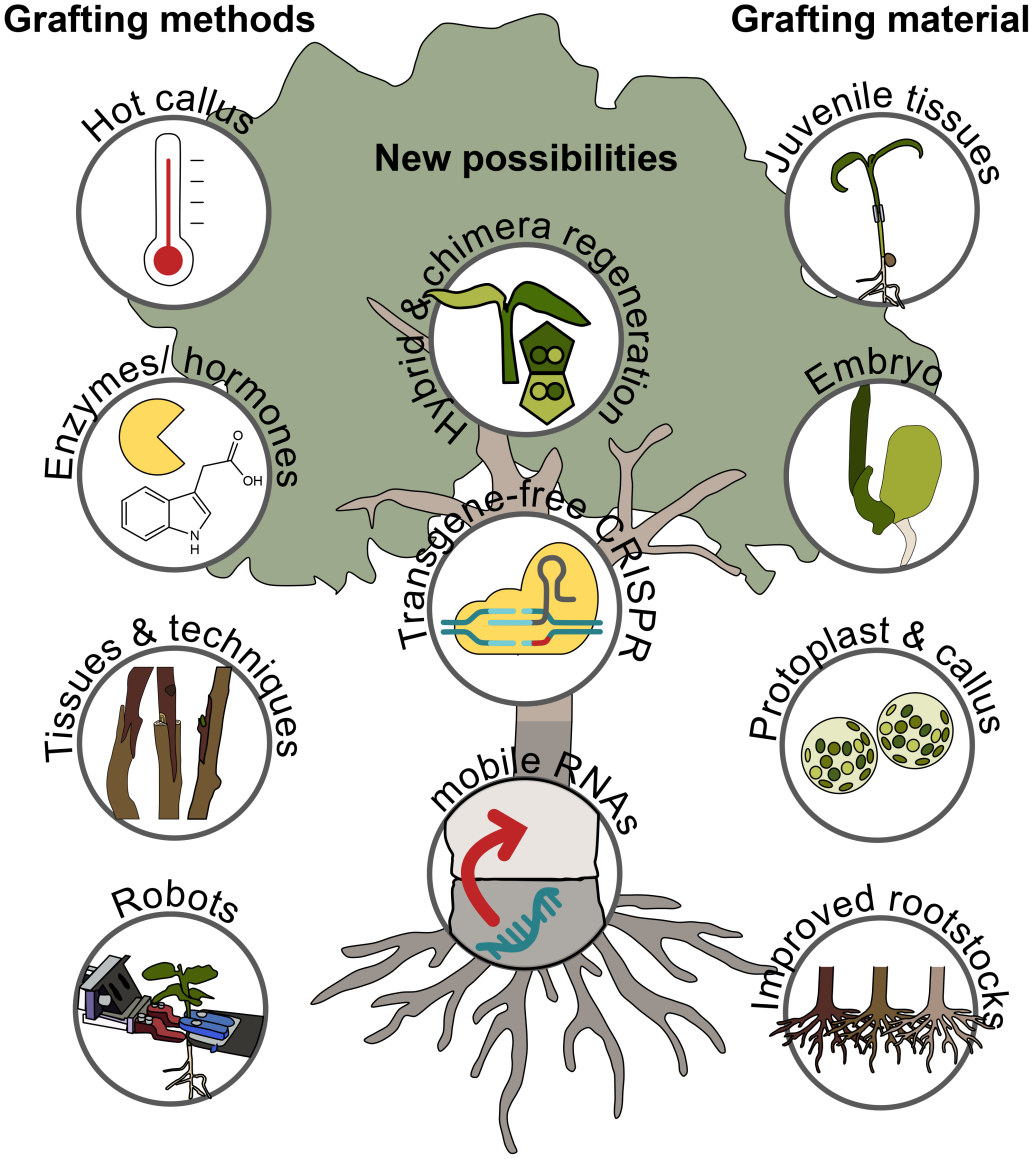
Grafting methods, materials and new possibilities. Different methods have been developed to improve grafting success. At the same time, the use of different grafting materials have expanded the range of plants that can be grafted and the phenotypes that can be accomplished. In the future, hybrid and chimera regeneration as well as the use of transgene‐free CRISPR offers new possibilities to produce even more desirable plants.

Although Darwin proposed his idea of graft hybridization in 1868, there was little evidence that true hybridization occurred at the graft junction until recently. Grafts made between *Nicotiana tabacum* containing different antibiotic selection markers in the scion and rootstock allowed the isolation of shoots formed at the graft junction containing both antibiotic markers (Stegemann & Bock, 2009). Such plants had cells with DNA from both graft partners and plants were recovered at rates slightly higher than one hybrid per graft junction (Stegemann & Bock, 2009). Further studies revealed the transfer of chloroplasts, mitochondria and even the whole genomes occurred (Fuentes et al., 2014; Gurdon et al., 2016; Stegemann et al., 2012; Thyssen et al., 2012). The transfer of the nuclear genome between *N. tabacum* and *N. glauca* allowed the formation of tetraploid cells at the graft junction that could then be isolated using antibiotic selection and grown into a novel species (Fuentes et al., 2014). This method of asexual hybridization thus presents a promising way to combine genomes from different species that cannot be normally hybridized yet can be grafted. For example, the high value metabolite, ketocarotenoid astaxanthin, was transgenically expressed in *N. tabacum* plastids. These plants were then grafted to *Nicotiana glauca* to transfer the chloroplasts and allow astaxanthin production in a nicotine‐free species (Lu et al., 2017). However, important outstanding questions and technical limitations remains. It is unclear exactly how the DNA transfers. Plastids have been observed moving cell‐to‐cell through 1.5 μm intercellular pores at the junction (Hertle et al., 2021). However, these data do not rule out other mechanisms given that the same organelle moving across the junction in real time has not yet been observed and other organelles, such as nuclei, are much larger and potentially harder to move through a narrow pore (Chambaud et al., 2022; Hertle et al., 2021). It is also possible that a process similar to protoplast fusion, which can also cause cell hybridization, occurs at the graft junction from weakened cell walls (Chambaud et al., 2022; Hertle et al., 2021). One limitation for this hybridization technology is that antibiotic selective markers are used to select for these rare events, perhaps as low as one hybrid cell per junction (Stegemann & Bock, 2009). Techniques are needed to improve rates of hybrid formation and to avoid the use of transgenes, allowing this technology to be used more widely (Figure 3).

## RNA MOBILITY TO ENGINEER TRAITS

The mobility of RNAs across the graft junction has allowed multiple engineering possibilities (Figure 3). By engineering rootstocks with transgenes that generate small interfering RNAs targeting Prunus necrotic ringspot virus or potato spindle tuber viroid, resistance to these viruses was enhanced in non‐transgenic cherry or tobacco scions, respectively (Kasai et al., 2013; Zhao & Song, 2014). Similarly, rootstocks expressing siRNAs to the meiosis factor *DISRUPTED MEIOTIC cDNA 1* (*DMC1*) resulted in siRNAs moving into the flowers and causing partial sterility in *N. tabacum* (Zhang et al., 2014). Transgenic *N. benthamiana* rootstocks expressing siRNAs caused epigenetic modifications in potato scions that were heritable to tubers (Kasai et al., 2016). Engineering viruses to silence genes, and then using these in grafting experiments can also improve disease resistance in the scion (Krueger et al., 2024). Several thousand mRNAs also move systemically between shoot and root and a tRNA‐like structure (TLS) was enriched in these mobile RNAs (Zhang et al., 2016). Adding this motif to a non‐mobile RNA conferred mobility, indicating that the TLS was sufficient to confer mobility across a graft junction in Arabidopsis and tobacco (Zhang et al., 2016). Fusing a TLS to a Cas9 mRNA and CRISPR guide RNA created a mobile CRISPR‐Cas9 editing system, and through grafting, transgene RNAs moved from the transgenic rootstock to the wild‐type scion and caused heritable genome editing in the progeny (Yang et al., 2023). Such editing appeared in 0.5% of the progeny and this heritability has so far only been demonstrated in Arabidopsis (Yang et al., 2023). Thus, such as a system is highly promising for generating transgene‐free CRISPR‐Cas9 edits but further work is needed. This system relies on the conserved mobility of the TLS‐fusion RNAs in other species, on their editing ability and on our ability to graft. Testing the system in species beyond Arabidopsis should be a priority. Recently, *N. benthamiana* and Petunia were demonstrated to be nearly universally successful graft partners with a wide range of eudicots (Kurotani et al., 2022; Notaguchi et al., 2020). Generating such a mobile gene editing system in *N. benthamiana* and using this species as a RNA delivery system to genome edit various species via grafting could be a promising technological development.

## THE FUTURE OF GRAFT ENGINEERING

Plant grafting has existed since antiquity but its recent use continues to grow with new species, new techniques, and new applications appearing (Table 1; Figure 3). Major challenges remain with grafting and the use of grafted plants. Costs remain high and graft failure rates remain a problem for the industry (Barrett et al., 2012; Li, Han, et al., 2021). Furthermore, the mechanistic basis for many of the graft‐induced traits such as dwarfing remain unknown. Efforts are needed to lower the costs associated with grafted material and to improve the benefits of rootstocks through dedicated breeding programs, thus helping justify higher costs. Automation of micrografted material should also be prioritized to rapidly and efficiently graft given that young materials graft so efficiently (Feng et al., 2024; Reeves et al., 2022). Identifying markers associated with graft incompatibility would help diagnose this phenomenon and allow breeding to avoid genes and loci that cause graft failure. Understanding the mechanistic basis for incompatibility would also help develop treatments to improve graft success rates. There is also a pressing need to better understand the basis for both autonomous and non‐autonomous grafting‐induced traits (Box 2).

More recent applications for grafting, such as graft chimeras, graft hybrids and mobile systemic RNAs show promise as novel ways to generate new species or new genotypes for agriculture and horticulture. Further work is needed to better understand how these combinations form, their stability, their efficiency, and the heritability of editing or epigenome modifications. We are now able to graft more species than ever, in particular with the development of monocot grafting (Reeves et al., 2022). The use of young tissues and *Nicotiana* has also allowed us to overcome grafting barriers for many species (Feng et al., 2024; Notaguchi et al., 2020; Reeves et al., 2022). Thus, combining novel applications such as chimeras, hybrids and mobile RNAs with these new grafting techniques should allow a transformation in grafting biology and the development of new species and new technologies. Given the rapid progress and interest in grafting the past 20 years, the future for grafting research looks promising and the potential applications will allow for major improvements in science, horticulture and agriculture.

## CONFLICT OF INTEREST

The authors declares no conflict of interest.

## Data Availability

Data sharing not applicable to this article as no datasets were generated or analysed during the current study.

## References

[tpj70057-bib-0001] Ahsan, M.U. , Hayward, A. , Alam, M. , Bandaralage, J.H. , Topp, B. , Beveridge, C.A. et al. (2019) Scion control of miRNA abundance and tree maturity in grafted avocado. BMC Plant Biology, 19, 382. 31481026 10.1186/s12870-019-1994-5 PMC6724330

[tpj70057-bib-0002] Almqvist, C. & Ekberg, I. (2001) Interstock and GA4/7 effects on flowering after topgrafting in *Pinus sylvestris* . Forest Genetics, 8, 279–284.

[tpj70057-bib-0003] Andersen, T.G. , Nour‐Eldin, H.H. , Fuller, V.L. , Olsen, C.E. , Burow, M. & Halkier, B.A. (2013) Integration of biosynthesis and long‐distance transport establish organ‐specific glucosinolate profiles in vegetative Arabidopsis. Plant Cell, 25, 3133–3145. 23995084 10.1105/tpc.113.110890 PMC3784604

[tpj70057-bib-0004] Atkinson, C.J. , Else, M.A. , Taylor, L. & Dover, C.J. (2003) Root and stem hydraulic conductivity as determinants of growth potential in grafted trees of apple (*Malus pumila* Mill.). Journal of Experimental Botany, 54, 1221–1229. 12654873 10.1093/jxb/erg132

[tpj70057-bib-0005] Avanzato, D. & Tamponi, G. (1988) The effect of heating of walnut graft unions on grafting success. Acta Horticulturae, 227, 79–83.

[tpj70057-bib-0006] Balfagón, D. , Rambla, J.L. , Granell, A. , Arbona, V. & Gómez‐Cadenas, A. (2022) Grafting improves tolerance to combined drought and heat stresses by modifying metabolism in citrus scion. Environmental and Experimental Botany, 195, 104793.

[tpj70057-bib-0007] Barrett, C.E. , Zhao, X. & Hodges, A.W. (2012) Cost benefit analysis of using grafted transplants for root‐knot nematode management in organic heirloom tomato production. HortTechnology, 22, 252–257.

[tpj70057-bib-0008] Bartusch, K. & Melnyk, C.W. (2020) Insights into plant surgery: an overview of the multiple grafting techniques for *Arabidopsis thaliana* . Frontiers in Plant Science, 11, 613442. 33362838 10.3389/fpls.2020.613442 PMC7758207

[tpj70057-bib-0009] Bhogale, S. , Mahajan, A.S. , Natarajan, B. , Rajabhoj, M. , Thulasiram, H.V. & Banerjee, A.K. (2013) MicroRNA156: a potential graft‐transmissible microRNA that modulates plant architecture and tuberization in *Solanum tuberosum* ssp. *andigena* . Plant Physiology, 164, 1011–1027. 24351688 10.1104/pp.113.230714 PMC3912076

[tpj70057-bib-0010] Binding, H. , Witt, D. , Monzer, J. , Mordhorst, G. & Kollmann, R. (1987) Plant cell graft chimeras obtained by co‐culture of isolated protoplasts. Protoplasma, 141, 64–73.

[tpj70057-bib-0011] Bruijn, G.D. & Dharmaputra, T.S. (1974) Mukibat system, a high yielding method of cassava production in Indonesia. Netherlands Journal of Agricultural Science, 22, 89–100.

[tpj70057-bib-0012] Burge, G.K. , Morgan, E.R. & Seelye, J.F. (2002) Opportunities for synthetic plant chimeral breeding: past and future. Plant Cell, Tissue and Organ Culture, 70, 13–21.

[tpj70057-bib-0013] Calderwood, A. , Kopriva, S. & Morris, R.J. (2016) Transcript abundance explains mRNA mobility data in *Arabidopsis thaliana* . Plant Cell, 28, 610–615. 26952566 10.1105/tpc.15.00956 PMC4826013

[tpj70057-bib-0014] Cerruti, E. , Gisbert, C. , Drost, H.‐G. , Valentino, D. , Portis, E. , Barchi, L. et al. (2021) Grafting vigour is associated with DNA de‐methylation in eggplant. Horticulture Research, 8, 241. 34719687 10.1038/s41438-021-00660-6 PMC8558322

[tpj70057-bib-0015] Chambaud, C. , Cookson, S.J. , Ollat, N. , Bayer, E. & Brocard, L. (2022) A correlative light electron microscopy approach reveals plasmodesmata ultrastructure at the graft interface. Plant Physiology, 188, 44–55. 34687300 10.1093/plphys/kiab485 PMC8774839

[tpj70057-bib-0016] Chen, L.‐P. , Ge, Y.‐M. & Zhu, X.‐Y. (2006) Artificial synthesis of interspecific chimeras between tuber mustard (*Brassica juncea*) and cabbage (*Brassica oleracea*) and cytological analysis. Plant Cell Reports, 25, 907–913. 16565861 10.1007/s00299-006-0150-5

[tpj70057-bib-0017] Chen, Y. , Liang, Z. , Krstic, M. , Clingeleffer, P. , Howell, K. , Chen, D. et al. (2024) The influences of rootstock on the performance of pinot noir (*Vitis vinifera* L.): berry and wine composition. Australian Journal of Grape and Wine Research, 2024, 7586202.

[tpj70057-bib-0018] Cohen, R. , Burger, Y. , Horev, C. , Koren, A. & Edelstein, M. (2007) Introducing grafted cucurbits to modern agriculture: the Israeli experience. Plant Disease, 91, 916–923. 30780423 10.1094/PDIS-91-8-0916

[tpj70057-bib-0019] Cookson, S.J. & Ollat, N. (2013) Grafting with rootstocks induces extensive transcriptional re‐programming in the shoot apical meristem of grapevine. BMC Plant Biology, 13, 147. 24083813 10.1186/1471-2229-13-147 PMC3852942

[tpj70057-bib-0020] Corbesier, L. , Vincent, C. , Jang, S. , Fornara, F. , Fan, Q. , Searle, I. et al. (2007) FT protein movement contributes to long‐distance signaling in floral induction of Arabidopsis. Science, 316, 1030–1033. 17446353 10.1126/science.1141752

[tpj70057-bib-0021] Darwin, C. (1868) The variation of animals and plants under domestication. London: J. murray. PMC5165317 30163123

[tpj70057-bib-0022] de Oliveira Castro, C.A. , dos Santos, G.A. , Takahashi, E.K. , Pires Nunes, A.C. , Souza, G.A. & de Resende, M.D.V. (2021) Accelerating eucalyptus breeding strategies through top grafting applied to young seedlings. Industrial Crops and Products, 171, 113906.

[tpj70057-bib-0023] Dong, D. , Shi, Y.‐N. , Mou, Z.‐M. , Chen, S.‐Y. & Zhao, D.‐K. (2022) Grafting: a potential method to reveal the differential accumulation mechanism of secondary metabolites. Horticulture Research, 9, uhac050. 10.1093/hr/uhac050 35591927 PMC9113227

[tpj70057-bib-0024] Errea, P. , Felipe, A. & Herrero, M. (1994) Graft establishment between compatible and incompatible *Prunus* spp. Journal of Experimental Botany, 45, 393–401.

[tpj70057-bib-0025] Feng, M. , Zhang, A. , Nguyen, V. , Bisht, A. , Almqvist, C. , De Veylder, L. et al. (2024) A conserved graft formation process in Norway spruce and Arabidopsis identifies the PAT gene family as central regulators of wound healing. Nature Plants, 10, 53–65. 38168607 10.1038/s41477-023-01568-w PMC10808061

[tpj70057-bib-0026] Filippis, I. , Lopez‐Cobollo, R. , Abbott, J. , Butcher, S. & Bishop, G.J. (2013) Using a periclinal chimera to unravel layer‐specific gene expression in plants. The Plant Journal, 75, 1039–1049. 23725542 10.1111/tpj.12250 PMC4223383

[tpj70057-bib-0027] Foster, T.M. , Celton, J.M. , Chagne, D. , Tustin, D.S. & Gardiner, S.E. (2015) Two quantitative trait loci, Dw1 and Dw2, are primarily responsible for rootstock‐induced dwarfing in apple. Horticulture Research, 2, 15001. 26504562 10.1038/hortres.2015.1 PMC4595989

[tpj70057-bib-0028] Foster, T.M. , McAtee, P.A. , Waite, C.N. , Boldingh, H.L. & McGhie, T.K. (2017) Apple dwarfing rootstocks exhibit an imbalance in carbohydrate allocation and reduced cell growth and metabolism. Horticulture Research, 4, 17009. 28435686 10.1038/hortres.2017.9 PMC5381684

[tpj70057-bib-0029] Frank, M.H. & Chitwood, D.H. (2016) Plant chimeras: the good, the bad, and the ‘Bizzaria’. Developmental Biology, 419, 41–53. 27381079 10.1016/j.ydbio.2016.07.003

[tpj70057-bib-0030] Fuentes, I. , Stegemann, S. , Golczyk, H. , Karcher, D. & Bock, R. (2014) Horizontal genome transfer as an asexual path to the formation of new species. Nature, 511, 232–235. 24909992 10.1038/nature13291

[tpj70057-bib-0031] Garcia‐Lozano, M. , Dutta, S.K. , Natarajan, P. , Tomason, Y.R. , Lopez, C. , Katam, R. et al. (2020) Transcriptome changes in reciprocal grafts involving watermelon and bottle gourd reveal molecular mechanisms involved in increase of the fruit size, rind toughness and soluble solids. Plant Molecular Biology, 102, 213–223. 31845303 10.1007/s11103-019-00942-7

[tpj70057-bib-0032] Garner, R.J. & Bradley, S. (2013) The grafter's handbook. London: Mitchell Beazley.

[tpj70057-bib-0033] Goffreda, J.C. , Szymkowiak, E.J. , Sussex, I.M. & Mutschler, M.A. (1990) Chimeric tomato plants show that aphid resistance and triacylglucose production are epidermal autonomous characters. Plant Cell, 2, 643–649. 2136638 10.1105/tpc.2.7.643 PMC159918

[tpj70057-bib-0034] Grieneisen, M.L. , Aegerter, B.J. , Scott Stoddard, C. & Zhang, M. (2018) Yield and fruit quality of grafted tomatoes, and their potential for soil fumigant use reduction. A meta‐analysis. Agronomy for Sustainable Development, 38, 29.

[tpj70057-bib-0035] Grubb, N.H. (1939) The influence of intermediate stem‐pieces in double‐worked apple and pear trees. Scientific Horticulture, 7, 17–23.

[tpj70057-bib-0036] Gurdon, C. , Svab, Z. , Feng, Y. , Kumar, D. & Maliga, P. (2016) Cell‐to‐cell movement of mitochondria in plants. Proceedings of the National Academy of Sciences of the United States of America, 113, 3395–3400. 26951647 10.1073/pnas.1518644113 PMC4812711

[tpj70057-bib-0037] Harrison, N. , Harrison, R.J. , Barber‐Perez, N. , Cascant‐Lopez, E. , Cobo‐Medina, M. , Lipska, M. et al. (2016) A new three‐locus model for rootstock‐induced dwarfing in apple revealed by genetic mapping of root bark percentage. Journal of Experimental Botany, 67, 1871–1881. 26826217 10.1093/jxb/erw001 PMC4783367

[tpj70057-bib-0038] Hasbioğlu, Y. , Weber, S. , Kragler, F. & Machin, F.Q. (2023) Arabidopsis callus grafts: a system to study symplasmic intercellular macromolecular transport and gene silencing spread. The Plant Journal, 115, 301–316. 37243907 10.1111/tpj.16326

[tpj70057-bib-0039] Hatton, R.G. (1939) Rootstock work at East Malling. Scientific Horticulture, 7, 7–16.

[tpj70057-bib-0040] Hertle, A.P. , Haberl, B. & Bock, R. (2021) Horizontal genome transfer by cell‐to‐cell travel of whole organelles. Science Advances, 7(1), eabd8215. 10.1126/sciadv.abd8215 33523859 PMC7775762

[tpj70057-bib-0041] Heuchel, A. , Hall, D. , Almqvist, C. , Wennström, U. & Persson, T. (2024) Topgrafting as a tool in operational scots pine breeding. Journal of Forestry Research, 35, 111.

[tpj70057-bib-0042] Hmmam, I. , Abdelaal, R.A. & Gomaa, A.H. (2023) Insight into chilling stress response of key citrus grafting combinations grown in Egypt. Plant Stress, 8, 100155.

[tpj70057-bib-0043] Huang, L.‐C. , Lius, S. , Huang, B.‐L. , Murashige, T. , Mahdi, E.F.M. & Van Gundy, R. (1992) Rejuvenation of *Sequoia sempervirens* by repeated grafting of shoot tips onto juvenile rootstocks in vitro: model for phase reversal of trees. Plant Physiology, 98, 166–173. 16668609 10.1104/pp.98.1.166 PMC1080165

[tpj70057-bib-0044] Jayawickrama, K.J.S. , Jett, J.B. & McKeand, S.E. (1991) Rootstock effects in grafted conifers: a review. New Forests, 5, 157–173.

[tpj70057-bib-0045] Jonard, R. (1986) Micrografting and its applications to tree improvement. In: Bajaj, Y.P.S. (Ed.) Trees I. Berlin, Heidelberg: Springer, pp. 31–48.

[tpj70057-bib-0046] Jørgensen, C.A. & Crane, M.B. (1927) Formation and morphology of *Solanum* chimaeras. Journal of Genetics, 18, 247–273.

[tpj70057-bib-0047] Kappel, N. , Palla, B. , Challa, L. & Mozafarian, M. (2024) Rootstock and scion anatomical parameters in grafted eggplant seedlings, influencing growth and fruit production. BMC Plant Biology, 24, 1207. 39701977 10.1186/s12870-024-05926-4 PMC11656800

[tpj70057-bib-0048] Kasai, A. , Bai, S. , Hojo, H. & Harada, T. (2016) Epigenome editing of potato by grafting using transgenic tobacco as siRNA donor. PLoS One, 11, e0161729. 27564864 10.1371/journal.pone.0161729 PMC5001710

[tpj70057-bib-0049] Kasai, A. , Sano, T. & Harada, T. (2013) Scion on a stock producing siRNAs of potato spindle tuber viroid (PSTVd) attenuates accumulation of the viroid. PLoS One, 8, e57736. 23469061 10.1371/journal.pone.0057736 PMC3585205

[tpj70057-bib-0050] Kawakatsu, Y. , Sawai, Y. , Kurotani, K.I. , Shiratake, K. & Notaguchi, M. (2020) An in vitro grafting method to quantify mechanical forces of adhering tissues. Plant Biotechnology (Tokyo), 37, 451–458. 10.5511/plantbiotechnology.20.0925a PMC8034679 33850433

[tpj70057-bib-0051] King, S.R. , Davis, A.R. , Liu, W. & Levi, A. (2008) Grafting for disease resistance. HortScience, 43, 1673–1676.

[tpj70057-bib-0052] King, S.R. , Davis, A.R. , Zhang, X. & Crosby, K. (2010) Genetics, breeding and selection of rootstocks for Solanaceae and Cucurbitaceae. Scientia Horticulturae, 127, 106–111.

[tpj70057-bib-0053] Knäbel, M. , Friend, A.P. , Palmer, J.W. , Diack, R. , Wiedow, C. , Alspach, P. et al. (2015) Genetic control of pear rootstock‐induced dwarfing and precocity is linked to a chromosomal region syntenic to the apple Dw1 loci. BMC Plant Biology, 15, 230. 26394845 10.1186/s12870-015-0620-4 PMC4580296

[tpj70057-bib-0054] Korba, J. , Patáková, K. & Kůdela, V. (2002) Effect of rootstock clones on fire blight susceptibility in scion apple cultivars. Plant Protection Science, 38, 552–554.

[tpj70057-bib-0055] Köse, C. & Güleryüz, M. (2006) Effects of auxins and cytokinins on graft union of grapevine (*Vitis vinifera*). New Zealand Journal of Crop and Horticultural Science, 34, 145–150.

[tpj70057-bib-0056] Krueger, R.R. , Chen, A.Y.S. , Zhou, J.S. , Liu, S. , Xu, H.K. & Ng, J.C.K. (2024) An engineered citrus tristeza virus (T36CA)‐based vector induces gene‐specific RNA silencing and is graft transmissible to commercial citrus varieties. Phytopathology, 114, 2453–2462. 39115802 10.1094/PHYTO-05-24-0167-R

[tpj70057-bib-0057] Külla, T. & Lõhmus, K. (1999) Influence of cultivation method on root grafting in Norway spruce (*Picea abies* (L.) Karst.). Plant and Soil, 217, 91–100.

[tpj70057-bib-0058] Kumari, A. , Kumar, J. , Kumar, A. , Chaudhury, A. & Singh, S.P. (2015) Grafting triggers differential responses between scion and rootstock. PLoS One, 10, e0124438. 25874958 10.1371/journal.pone.0124438 PMC4395316

[tpj70057-bib-0059] Kundariya, H. , Yang, X. , Morton, K. , Sanchez, R. , Axtell, M.J. , Hutton, S.F. et al. (2020) MSH1‐induced heritable enhanced growth vigor through grafting is associated with the RdDM pathway in plants. Nature Communications, 11, 5343. 10.1038/s41467-020-19140-x PMC7582163 33093443

[tpj70057-bib-0060] Kurotani, K.‐I. , Huang, C. , Okayasu, K. , Suzuki, T. , Ichihashi, Y. , Shirasu, K. et al. (2022) Discovery of the interfamily grafting capacity of Petunia, a floricultural species. Horticulture Research, 9, uhab056. 10.1093/hr/uhab056 35048114 PMC8969063

[tpj70057-bib-0061] Kyriacou, M.C. , Rouphael, Y. , Colla, G. , Zrenner, R. & Schwarz, D. (2017) Vegetable grafting: the implications of a growing agronomic imperative for vegetable fruit quality and nutritive value. Frontiers in Plant Science, 8, 741. 10.3389/fpls.2017.00741 28553298 PMC5427113

[tpj70057-bib-0062] Lee, C. , Harvey, J.T. , Nagila, A. , Qin, K. & Leskovar, D.I. (2023) Thermotolerance of tomato plants grafted onto wild relative rootstocks. Frontiers in Plant Science, 14, 1252456. 10.3389/fpls.2023.1252456 38053760 PMC10694270

[tpj70057-bib-0063] Lee, J. , Kubota, C. , Tsao, S.J. , Bie, Z. , Echevarria, P.H. , Morra, L. et al. (2010) Current status of vegetable grafting: diffusion, grafting techniques, automation. Scientia Horticulturae, 127, 93–105.

[tpj70057-bib-0064] Lee, J.‐M. (1994) Cultivation of grafted vegetables I. Current status, grafting methods, and benefits. HortScience, 29, 235–239.

[tpj70057-bib-0065] Lev‐Yadun, S. & Sprugel, D. (2011) Why should trees have natural root grafts? Tree Physiology, 31, 575–578. 21778291 10.1093/treephys/tpr061

[tpj70057-bib-0066] Li, D. , Han, F. , Liu, X. , Lv, H. , Li, L. , Tian, H. et al. (2021) Localized graft incompatibility in kiwifruit: analysis of homografts and heterografts with different rootstock & scion combinations. Scientia Horticulturae, 283, 110080.

[tpj70057-bib-0067] Li, J. , Wang, Y. , Zhang, L. , Liu, B. , Cao, L. , Qi, Z. et al. (2013) Heritable variation and small RNAs in the progeny of chimeras of *Brassica juncea* and *Brassica oleracea* . Journal of Experimental Botany, 64, 4851–4862. 24006424 10.1093/jxb/ert266 PMC3830474

[tpj70057-bib-0068] Li, J.‐X. , Rao, L.‐L. , Xie, H. , Schreiner, M. , Chen, L.‐P. & Liu, Y.‐Q. (2017) Morphology and glucosinolate profiles of chimeric brassica and the responses of *Bemisia tabaci* in host selection, oviposition and development. Journal of Integrative Agriculture, 16, 2009–2018.

[tpj70057-bib-0069] Li, S. , Wang, X. , Xu, W. , Liu, T. , Cai, C. , Chen, L. et al. (2021) Unidirectional movement of small RNAs from shoots to roots in interspecific heterografts. Nature Plants, 7, 50–59. 33452489 10.1038/s41477-020-00829-2

[tpj70057-bib-0070] Li, W. , Chu, C. , Li, H. , Zhang, H. , Sun, H. , Wang, S. et al. (2024) Near‐gapless and haplotype‐resolved apple genomes provide insights into the genetic basis of rootstock‐induced dwarfing. Nature Genetics, 56, 505–516. 38347217 10.1038/s41588-024-01657-2

[tpj70057-bib-0071] Li, Y. & Zhao, D. (2021) Transcriptome analysis of scions grafted to potato rootstock for improving late blight resistance. BMC Plant Biology, 21, 272. 34130637 10.1186/s12870-021-03039-w PMC8204497

[tpj70057-bib-0131] Liu, Y. (2018) Darwin's Pangenesis and Graft Hybridization. Advances in Genetics, 102:27–66. 30122234 10.1016/bs.adgen.2018.05.007

[tpj70057-bib-0072] Liang, H. , Hu, Y. , Pang, W. , Liu, W. & Yang, M. (2011) Studies on kiwifruit improvement by multiple top grafting. Acta Horticulturae, 913, 365–371.

[tpj70057-bib-0073] Liu, N. , Yang, J. , Fu, X. , Zhang, L. , Tang, K. , Guy, K.M. et al. (2016) Genome‐wide identification and comparative analysis of grafting‐responsive mRNA in watermelon grafted onto bottle gourd and squash rootstocks by high‐throughput sequencing. Molecular Genetics and Genomics, 291, 621–633. 26500104 10.1007/s00438-015-1132-5

[tpj70057-bib-0074] Lordan, J. , Gomez, M. , Francescatto, P. & Robinson, T.L. (2019) Long‐term effects of tree density and tree shape on apple orchard performance, a 20 year study – part 2, economic analysis. Scientia Horticulturae, 244, 435–444.

[tpj70057-bib-0075] Lu, Y. , Stegemann, S. , Agrawal, S. , Karcher, D. , Ruf, S. & Bock, R. (2017) Horizontal transfer of a synthetic metabolic pathway between plant species. Current Biology, 27, 3034–3041.e3. 28943084 10.1016/j.cub.2017.08.044

[tpj70057-bib-0076] Lucas, W.J. , Groover, A. , Lichtenberger, R. , Furuta, K. , Yadav, S.‐R. , Helariutta, Y. et al. (2013) The plant vascular system: evolution, development and functions. Journal of Integrative Plant Biology, 55, 294–388. 23462277 10.1111/jipb.12041

[tpj70057-bib-0077] Matsumoto‐Kitano, M. , Kusumoto, T. , Tarkowski, P. , Kinoshita‐Tsujimura, K. , Vaclavikova, K. , Miyawaki, K. et al. (2008) Cytokinins are central regulators of cambial activity. Proceedings of the National Academy of Sciences of the United States of America, 105, 20027–20031. 19074290 10.1073/pnas.0805619105 PMC2605004

[tpj70057-bib-0078] Melnyk, C.W. , Schuster, C. , Leyser, O. & Meyerowitz, E.M. (2015) A developmental framework for graft formation and vascular reconnection in Arabidopsis thaliana. Current Biology, 25, 1306–1318. 25891401 10.1016/j.cub.2015.03.032 PMC4798781

[tpj70057-bib-0079] Mudge, K. , Janick, J. , Scofield, S. & Goldschmidt, E.E. (2009) A history of grafting. New York: John Wiley & Sons, Inc.

[tpj70057-bib-0080] Murashige, T. , Bitters, W.P. , Rangan, T.S. , Nauer, E.M. , Roistacher, C.N. & Holliday, P.B. (1972) A technique of shoot apex grafting and its utilization towards recovering virus‐free citrus clones. HortScience, 7, 118–119.

[tpj70057-bib-0081] Musa, I. , Rafii, M.Y. , Ahmad, K. , Ramlee, S.I. , Md Hatta, M.A. , Oladosu, Y. et al. (2020) Effects of grafting on morphophysiological and yield characteristic of eggplant (*Solanum melongena* L.) grafted onto wild relative rootstocks. Plants, 9, 1583. 33203189 10.3390/plants9111583 PMC7696694

[tpj70057-bib-0082] Nassar, N.M. & Bomfim, N. (2013) Synthesis of periclinal chimera in cassava. Genetics and Molecular Research, 12, 610–617. 23512678 10.4238/2013.February.27.10

[tpj70057-bib-0083] Noguchi, T. , Hirata, Y. & Yagishita, N. (1992) Intervarietal and interspecific chimera formation by in vitro graft‐culture method in brassica. Theoretical and Applied Genetics, 83, 727–732. 24202747 10.1007/BF00226691

[tpj70057-bib-0084] Notaguchi, M. , Higashiyama, T. & Suzuki, T. (2014) Identification of mRNAs that move over long distances using an RNA‐seq analysis of *Arabidopsis*/*Nicotiana benthamiana* heterografts. Plant and Cell Physiology, 56, 311–321. 25527829 10.1093/pcp/pcu210

[tpj70057-bib-0085] Notaguchi, M. , Kurotani, K.I. , Sato, Y. , Tabata, R. , Kawakatsu, Y. , Okayasu, K. et al. (2020) Cell‐cell adhesion in plant grafting is facilitated by beta‐1,4‐glucanases. Science, 369, 698–702. 32764072 10.1126/science.abc3710

[tpj70057-bib-0086] Ntatsi, G. , Savvas, D. , Papasotiropoulos, V. , Katsileros, A. , Zrenner, R.M. , Hincha, D.K. et al. (2017) Rootstock sub‐optimal temperature tolerance determines transcriptomic responses after long‐term root cooling in rootstocks and scions of grafted tomato plants. Frontiers in Plant Science, 8, 911. 10.3389/fpls.2017.00911 28642763 PMC5462977

[tpj70057-bib-0087] Obolensky, G. (1960) Grafting of plant embryos and the use of ultrasonics. Qualitas Plantarum et Materiae Vegetabiles, 7, 273–288.

[tpj70057-bib-0088] Ohta, Y. & Van Chuong, P. (1975) Hereditary changes in *Capsicum annuum* L. I. Induced by ordinary grafting. Euphytica, 24, 355–368.

[tpj70057-bib-0089] Ollat, N. , Bordenave, L. , Tandonnet, J.P. , Boursiquot, J.M. & Marguerit, E. (2016) Grapevine rootstocks: origins and perspectives. Acta Horticulturae, 1136, 11–22.

[tpj70057-bib-0090] Ollat, N. , Tandonnet, J.P. , Lafontaine, M. & Schultz, H.R. (2003) Short and long term effects of three rootstocks on cabernet sauvignon vine behaviour and wine quality. Acta Horticulturae, 617, 95–99.

[tpj70057-bib-0091] Olmstead, M.A. , Lang, S.N. , Ewers, F.W. & Owens, S.A. (2006) Xylem vessel anatomy of sweet cherries grafted onto dwarfing and nondwarfing rootstocks. Journal of the American Society for Horticultural Science, 131, 577–585.

[tpj70057-bib-0092] Paajanen, P. , Tomkins, M. , Hoerbst, F. , Veevers, R. , Heeney, M. , Thomas, H.R. et al. (2024) Re‐analysis of mobile mRNA datasets highlights challenges in the detection of mobile transcripts from short‐read RNA‐seq data. *bioRxiv*. 2024.2007.2025.604588. 10.1101/2024.07.25.604588.

[tpj70057-bib-0093] Pant, B.D. , Buhtz, A. , Kehr, J. & Scheible, W.R. (2008) MicroRNA399 is a long‐distance signal for the regulation of plant phosphate homeostasis. The Plant Journal, 53, 731–738. 17988220 10.1111/j.1365-313X.2007.03363.x PMC2268993

[tpj70057-bib-0094] Rahemi, A. , Dodson Peterson, J.C. & Lund, K.T. (2022) Grape rootstocks breeding. In: Rahemi, A. , Dodson Peterson, J.C. & Lund, K.T. (Eds.) Grape rootstocks and related species. Cham: Springer International Publishing, pp. 23–30.

[tpj70057-bib-0095] Reeves, G. , Tripathi, A. , Singh, P. , Jones, M.R.W. , Nanda, A.K. , Musseau, C. et al. (2022) Monocotyledonous plants graft at the embryonic root–shoot interface. Nature, 602, 280–286. 34937943 10.1038/s41586-021-04247-y

[tpj70057-bib-0096] Regnault, T. , Davière, J.‐M. , Wild, M. , Sakvarelidze‐Achard, L. , Heintz, D. , Carrera Bergua, E. et al. (2015) The gibberellin precursor GA12 acts as a long‐distance growth signal in Arabidopsis. Nature Plants, 1, 15073. 27250008 10.1038/nplants.2015.73

[tpj70057-bib-0097] Rubio, B. , Stammitti, L. , Cookson, S.J. , Teyssier, E. & Gallusci, P. (2022) Small RNA populations reflect the complex dialogue established between heterograft partners in grapevine. Horticulture Research, 9, uhab067. 35048109 10.1093/hr/uhab067 PMC8935936

[tpj70057-bib-0098] Sakata, Y. , Ohara, T. & Sugiyama, M. (2007) The history and present state of the grafting of cucurbitaceous vegetables in Japan. Acta Horticulturae, 731, 159–170.

[tpj70057-bib-0099] Schulze, A. , Zimmer, M. , Mielke, S. , Stellmach, H. , Melnyk, C.W. , Hause, B. et al. (2019) Wound‐induced shoot‐to‐root relocation of JA‐Ile precursors coordinates Arabidopsis growth. Molecular Plant, 12, 1383–1394. 31181337 10.1016/j.molp.2019.05.013

[tpj70057-bib-0100] Seleznyova, A.N. , Tustin, D.S. & Thorp, T.G. (2008) Apple dwarfing rootstocks and Interstocks affect the type of growth units produced during the annual growth cycle: precocious transition to flowering affects the composition and vigour of annual shoots. Annals of Botany, 101, 679–687. 18263898 10.1093/aob/mcn007 PMC2710180

[tpj70057-bib-0101] Silva Souza, L. , Diniz, R.P. , Neves, R.D.J. , Alves, A.A.C. & Oliveira, E.J.D. (2018) Grafting as a strategy to increase flowering of cassava. Scientia Horticulturae, 240, 544–551. 30349150 10.1016/j.scienta.2018.06.070 PMC6039848

[tpj70057-bib-0102] Singh, H. , Kumar, P. , Kumar, A. , Kyriacou, M.C. , Colla, G. & Rouphael, Y. (2020) Grafting tomato as a tool to improve salt tolerance. Agronomy, 10, 263.

[tpj70057-bib-0103] Singh, J. , Fabrizio, J. , Desnoues, E. , Silva, J.P. , Busch, W. & Khan, A. (2019) Root system traits impact early fire blight susceptibility in apple (*Malus* × *domestica*). BMC Plant Biology, 19, 579. 31870310 10.1186/s12870-019-2202-3 PMC6929320

[tpj70057-bib-0104] Song, G.‐Q. , Walworth, A. , Lin, T. , Chen, Q. , Han, X. , Irina Zaharia, L. et al. (2019) VcFT‐induced mobile florigenic signals in transgenic and transgrafted blueberries. Horticulture Research, 6, 105. 31645960 10.1038/s41438-019-0188-5 PMC6804590

[tpj70057-bib-0105] Soumelidou, K. , Battey, N.H. , John, P. & Barnett, J.R. (1994) The anatomy of the developing bud Union and its relationship to dwarfing in apple. Annals of Botany, 74, 605–611.

[tpj70057-bib-0106] Spanò, R. , Ferrara, M. , Gallitelli, D. & Mascia, T. (2020) The role of grafting in the resistance of tomato to viruses. Plants, 9, 1042. 32824316 10.3390/plants9081042 PMC7463508

[tpj70057-bib-0107] Spanò, R. , Mascia, T. , Kormelink, R. & Gallitelli, D. (2015) Grafting on a non‐transgenic tolerant tomato variety confers resistance to the infection of a Sw5‐breaking strain of tomato spotted wilt virus via RNA silencing. PLoS One, 10, e0141319. 26496695 10.1371/journal.pone.0141319 PMC4619829

[tpj70057-bib-0108] Stegemann, S. & Bock, R. (2009) Exchange of genetic material between cells in plant tissue grafts. Science, 324, 649–651. 19407205 10.1126/science.1170397

[tpj70057-bib-0109] Stegemann, S. , Keuthe, M. , Greiner, S. & Bock, R. (2012) Horizontal transfer of chloroplast genomes between plant species. Proceedings of the National Academy of Sciences of the United States of America, 109, 2434–2438. 22308367 10.1073/pnas.1114076109 PMC3289295

[tpj70057-bib-0110] Suchoff, D.H. , Louws, F.J. & Gunter, C.C. (2019) Yield and disease resistance for three bacterial wilt‐resistant tomato rootstocks. HortTechnology, 29, 330–337.

[tpj70057-bib-0111] Taller, J. , Hirata, Y. , Yagishita, N. , Kita, M. & Ogata, S. (1998) Graft‐induced genetic changes and the inheritance of several characteristics in pepper (*Capsicum annuum* L.). Theoretical and Applied Genetics, 97, 705–713.

[tpj70057-bib-0112] Thieme, C.J. , Rojas‐Triana, M. , Stecyk, E. , Schudoma, C. , Zhang, W. , Yang, L. et al. (2015) Endogenous Arabidopsis messenger RNAs transported to distant tissues. Nature Plants, 1, 15025. 27247031 10.1038/nplants.2015.25

[tpj70057-bib-0113] Thyssen, G. , Svab, Z. & Maliga, P. (2012) Cell‐to‐cell movement of plastids in plants. Proceedings of the National Academy of Sciences of the United States of America, 109, 2439–2443. 22308369 10.1073/pnas.1114297109 PMC3289365

[tpj70057-bib-0114] Tsaballa, A. , Athanasiadis, C. , Pasentsis, K. , Ganopoulos, I. , Nianiou‐Obeidat, I. & Tsaftaris, A. (2013) Molecular studies of inheritable grafting induced changes in pepper (*Capsicum annuum*) fruit shape. Scientia Horticulturae, 149, 2–8.

[tpj70057-bib-0115] Wang, H. , Zhou, P. , Zhu, W. & Wang, F. (2019) De novo comparative transcriptome analysis of genes differentially expressed in the scion of homografted and heterografted tomato seedlings. Scientific Reports, 9, 20240. 31882801 10.1038/s41598-019-56563-z PMC6934607

[tpj70057-bib-0116] Warschefsky, E.J. , Klein, L.L. , Frank, M.H. , Chitwood, D.H. , Londo, J.P. , von Wettberg, E.J. et al. (2016) Rootstocks: diversity, domestication, and impacts on shoot phenotypes. Trends in Plant Science, 21, 418–437. 26698413 10.1016/j.tplants.2015.11.008

[tpj70057-bib-0117] Winkler, H. (1907) Über Pfropfbastarde und pflanzliche Chimären. Berichte der Deutschen Botanischen Gesellschaft, 25, 568–576.

[tpj70057-bib-0118] Wu, R. , Wang, X. , Lin, Y. , Ma, Y. , Liu, G. , Yu, X. et al. (2013) Inter‐species grafting caused extensive and heritable alterations of DNA methylation in Solanaceae plants. PLoS One, 8, e61995. 23614002 10.1371/journal.pone.0061995 PMC3628911

[tpj70057-bib-0119] Xie, Z. , Gu, S. , Chu, Q. , Li, B. , Fan, K. , Yang, Y. et al. (2020) Development of a high‐productivity grafting robot for Solanaceae. International Journal of Agricultural and Biological Engineering, 13, 82–90.

[tpj70057-bib-0120] Yang, L. , Machin, F. , Wang, S. , Saplaoura, E. & Kragler, F. (2023) Heritable transgene‐free genome editing in plants by grafting of wild‐type shoots to transgenic donor rootstocks. Nature Biotechnology, 41, 958–967. 10.1038/s41587-022-01585-8 PMC10344777 36593415

[tpj70057-bib-0132] Yang L. , Perrera V. , Saplaoura E. , Apelt F. , Bahin M. , Kramdi A. et al. (2019). m5C Methylation Guides Systemic Transport of Messenger RNA over Graft Junctions in Plants. Current Biology. 29(15):2465‐2476.e5. 31327714 10.1016/j.cub.2019.06.042

[tpj70057-bib-0121] Yu, N. , Cao, L. , Yuan, L. , Zhi, X. , Chen, Y. , Gan, S. et al. (2018) Maintenance of grafting‐induced epigenetic variations in the asexual progeny of *Brassica oleracea* and *B. juncea* chimera. The Plant Journal, 96, 22–38. 30086201 10.1111/tpj.14058

[tpj70057-bib-0122] Zhang, G. , Zhou, J. , Song, J. , Guo, X. , Nie, X. & Guo, H. (2022) Grafting‐induced transcriptome changes and long‐distance mRNA movement in the potato/*Datura stramonium* heterograft system. Horticulture, Environment, and Biotechnology, 63, 229–238.

[tpj70057-bib-0123] Zhang, M. , Su, H. , Gresshoff, P.M. & Ferguson, B.J. (2021) Shoot‐derived miR2111 controls legume root and nodule development. Plant, Cell & Environment, 44, 1627–1641. 10.1111/pce.13992 33386621

[tpj70057-bib-0124] Zhang, W. , Kollwig, G. , Stecyk, E. , Apelt, F. , Dirks, R. & Kragler, F. (2014) Graft‐transmissible movement of inverted‐repeat‐induced siRNA signals into flowers. The Plant Journal, 80, 106–121. 25039964 10.1111/tpj.12622

[tpj70057-bib-0125] Zhang, W. , Thieme, C.J. , Kollwig, G. , Apelt, F. , Yang, L. , Winter, N. et al. (2016) tRNA‐related sequences trigger systemic mRNA transport in plants. Plant Cell, 28, 1237–1249. 27268430 10.1105/tpc.15.01056 PMC4944404

[tpj70057-bib-0126] Zhang, Z. , Cao, B. , Gao, S. & Xu, K. (2019) Grafting improves tomato drought tolerance through enhancing photosynthetic capacity and reducing ROS accumulation. Protoplasma, 256, 1013–1024. 30805718 10.1007/s00709-019-01357-3

[tpj70057-bib-0127] Zhao, D. & Song, G.Q. (2014) Rootstock‐to‐scion transfer of transgene‐derived small interfering RNAs and their effect on virus resistance in nontransgenic sweet cherry. Plant Biotechnology Journal, 12, 1319–1328. 25132092 10.1111/pbi.12243

[tpj70057-bib-0128] Zheng, X. , Zhao, Y. , Shan, D. , Shi, K. , Wang, L. , Li, Q. et al. (2018) Md9 overexpression confers intensive dwarfing in the M26 rootstock of apple by directly inhibiting brassinosteroid synthetase Md4 expression. The New Phytologist, 217, 1086–1098. 29165808 10.1111/nph.14891

[tpj70057-bib-0129] Zheng, Y. , Luo, L. , Gao, Z. , Liu, Y. , Chen, Q. , Kong, X. et al. (2019) Grafting induces flowering time and tuber formation changes in *Brassica* species involving FT signalling. Plant Biology, 21, 1031–1038. 31267637 10.1111/plb.13024

[tpj70057-bib-0130] Zhou, J. , Hirata, Y. , Nou, I.‐S. , Shiotani, H. & Ito, T. (2002) Interactions between different genotypic tissues in citrus graft chimeras. Euphytica, 126, 355–364.

